# Supplementary figures and images for: Genome-Wide Identification of the NF-Y Gene Family and Their Involvement in Bolting and Flowering in Flowering Chinese Cabbage
Source: Int J Mol Sci. 2023 Jul 25;24(15):11898. doi: 10.3390/ijms241511898 (PMC10418651; doi:10.3390/ijms241511898)

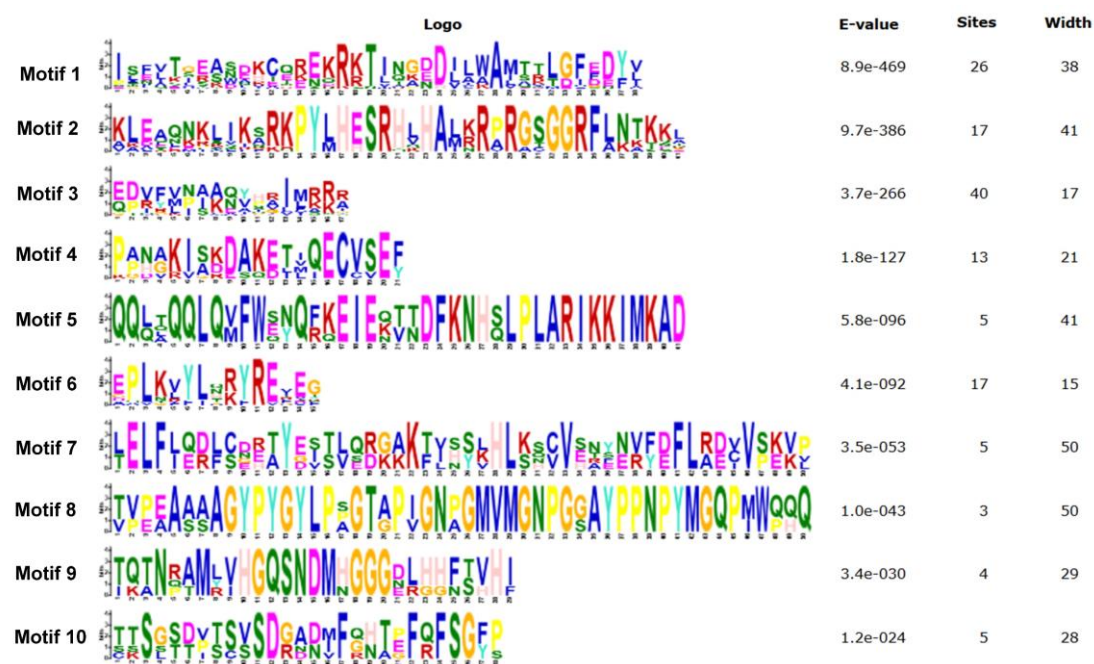

Figure S1. Sequence composition of motif.

Supplement: Supplementary file 1 [file ijms-24-11898-s001.zip › Figure S1.pdf]
